# Supplementary material for: Heterostructure particles enable omnidispersible in water and oil towards organic dye recycle
Source: Nat Commun. 2023 Sep 18;14:5779. doi: 10.1038/s41467-023-41053-8 (PMC10507067; doi:10.1038/s41467-023-41053-8)
Supplement: Supplementary file 1 — Supplementary information [file 41467_2023_41053_MOESM1_ESM.pdf]

Supplementary Information for

Heterostructure particles enable omnidispersible in water and oil towards organic dye recycle

Yongyang Song[1,2,†], Jiajia Zhou[3,†], Zhongpeng Zhu[4], Xiaoxia Li[1,2], Yue Zhang[1,2], Xinyi Shen[1,2], Padraic O'Reilly[5], Xiuling Li[6], Xinmiao Liang[6], Lei Jiang[1,2,4] & Shutao Wang[1,2,4]✉

[1] CAS Key Laboratory of Bio-inspired Materials and Interfacial Science, Technical Institute of Physics and Chemistry, Chinese Academy of Sciences, Beijing, P. R. China.

[2] University of Chinese Academy of Sciences, Beijing, P. R. China.

[3] South China Advanced Institute for Soft Matter Science and Technology, School of Emergent Soft Matter, South China University of Technology, Guangzhou, P. R. China.

[4] Suzhou Institute for Advanced Research, University of Science and Technology of China, Suzhou, P. R. China.

[5] Molecular Vista Inc., CA, USA.

[6] CAS Key Laboratory of Separation Science for Analytical Chemistry, Dalian Institute of Chemical Physics, Chinese Academy of Sciences, Dalian, P. R. China.

[†] These authors contributed equally: Yongyang Song, Jiajia Zhou.

✉e-mail: stwang@mail.ipc.ac.cn

Table of contents

1. Abbreviations......3

2. Chemicals......4

2.1 Chemicals for particle synthesis. 4

2.2 Solvents......4

2.3 Dyes. 4

3. Synthesis and characterizations of HL-HBPs. 5

3.1 Pore characterization of HL-HBPs. 5

3.2 Synthesis of HL-HBPs with varied charge. 6

3.3 Synthesis of HL-HBPs with varied particle diameter. 7

3.4 Synthesis of HL-HBPs with varied BET surface area. 8

4. Dispersion performance investigation......10

4.1 Characterizations of HLPs and HBPs. 10

4.2 Hydrophilicity/hydrophobicity nature of particles......11

5. Theoretical calculations for dispersion mechanism explanation. 13

5.1 Parameters for calculation......13

5.2 Demonstration of oil permeation into the HL-HBPs. 14

5.3 Refractive index prediction of PSS. 15

6. Calculation of the interaction sites. 16

7. Dye adsorption and desorption performance of HL-HBPs......17

7.1 Comparison of the dye adsorption performance. 17

7.2 Desorption of dyes from HL-HBPs characterized by LSCM. 19

7.3 Dye adsorption performance of HL-HBPs at different pH. 20

7.4 Comparison of dye adsorption kinetics......21

7.5 Property changes of HL-HBPs after adsorption-desorption. 22

7.6 Property changes of dyes after adsorption-desorption. 23

7.7 Property changes of membranes after filtration. 24

8. Large scale preparation of HL-HBPs. 25

9. Large scale dye recycle and wastewater treatment using HL-HBPs......26

10. Summary of solvent property. 27

11. Comparison of dye recycle performance. 28

References......29

1. Abbreviations.

Supplementary Table 1. Important abbreviations and corresponding full names.

Abbreviation	Full name
HL-HBPs	Hydrophilic-hydrophobic heterostructure particles
HLPs	Hydrophilic particles
HBPs	Hydrophobic particles
PSS	Poly(sodium 4-styrenesulfonate)
PSDVB	Poly(styrene-divinyl benzene)
PTMAEMC	Poly(2-trimethylammoniumethyl methacrylate chloride)
PiFM	Photo-induced force microscopy
SEM	Scanning electron microscopy
TEM	Transmission electron microscopy
AFM	Atomic force microscopy
LSCM	Laser scanning confocal microscopy
NMR	Nuclear magnetic resonance
BET	Brunauer-Emmett-Teller
MOFs	Metal organic frameworks
LDHs	Layered double hydroxides
DLVO	Derjaguin-Landau-Verwey-Overbeek
ζ	Zeta potential
η	Adsorption depth
κ^{-1}	Debye length

2. Chemicals.

2.1 Chemicals for particle synthesis.

SDS (99%), 1-chlorododecane (99%), St (99.5%), DVB (80%), SS (95%), and 2,2'-azoisobutyronitrile (99%) were purchased from J&K Scientific Ltd. (China). TMAEMC aqueous solution (75wt% in water) was ordered from Sigma Aldrich (USA). PVA (M_W = 20000-30000, hydrolysis degree = 88%) was purchased from Acros Organics (Belgium). Deionized water was prepared with Milli-Q system (18.2 MΩ·cm, Bedford, MA, USA).

2.2 Solvents.

ACN was ordered from Merck Corp. (Darmstadt, Germany). Toluene was ordered from Xilong Scientific (China). Octane and n-hexane were ordered from Aladdin (USA). Ethanol, DMSO, EA, and TCM were purchased from Sinopharm Chemical Reagents Co., Ltd. (China).

2.3 Dyes.

RB (95%), MG, MLB, CV, MV (75%), MB, and MO were purchased from J&K Scientific Ltd. (China). AF and EB were purchased from TCI (Japan). CR (98%) was purchased from Aladdin (USA).

3. Synthesis and characterizations of HL-HBPs.

3.1 Pore characterization of HL-HBPs.

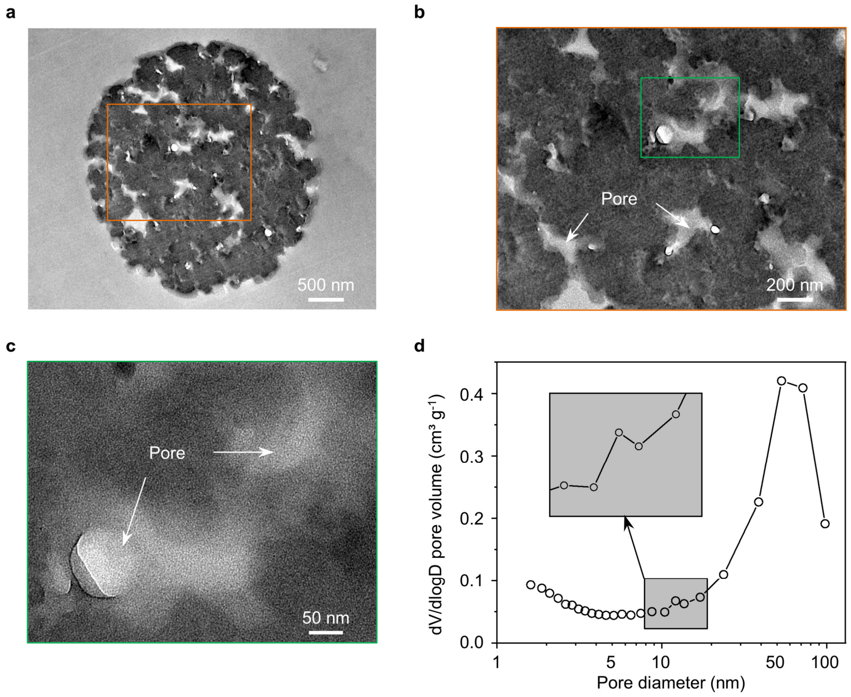

Supplementary Fig. 1. Pore characterization of the PSS-PSDVB HL-HBPs. a,**b**,**c**, Cross-section TEM images. **d**, Pore size distribution curve. Pore size distribution curve was obtained by nitrogen adsorption-desorption tests on a physisorption apparatus (Micromeritics, ASAP 2460), before which the particles were degassed at 80 ºC for 8 h.

3.2 Synthesis of HL-HBPs with varied charge.

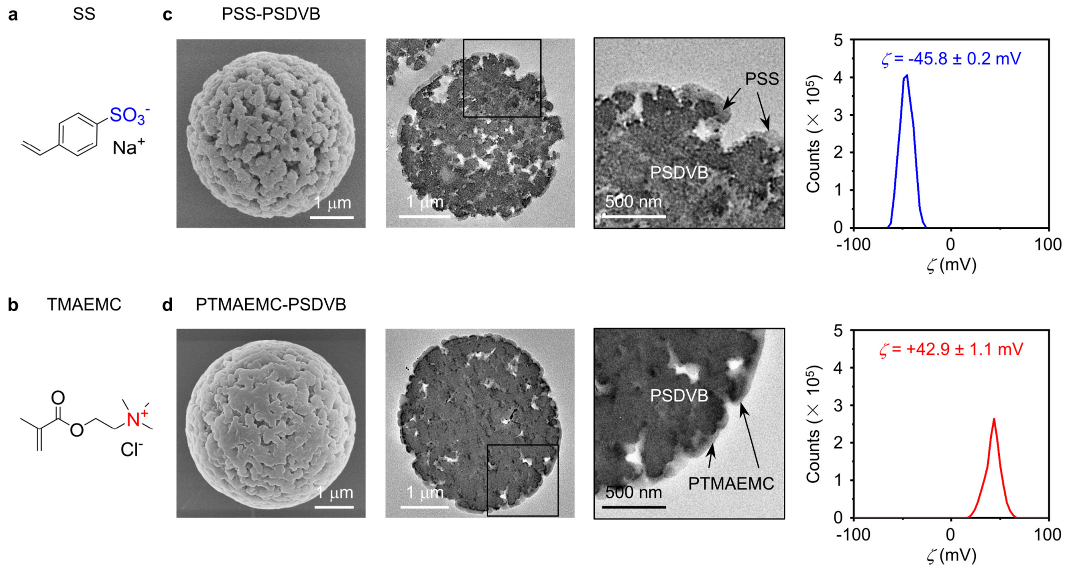

Supplementary Fig. 2. Synthesis and characterizations of HL-HBPs with negative charge and positive charge. a,**b**, Molecule structure of negatively charged hydrophilic monomer (SS) and positively charged hydrophilic monomer (TMAEMC) for the synthesis of HL-HBPs. **c**,**d**, SEM image, cross-section TEM images, and ζ distribution curve of negatively charged HL-HBPs (PSS-PSDVB) and positively charged HL-HBPs (PTMAEMC-PSDVB). The synthesis process of PSS-PSDVB and PTMAEMC-PSDVB is described in the main text. ζ (PSS-PSDVB) = -45.8 ± 0.2 mV, ζ (PTMAEMC-PSDVB) = +42.9 ± 1.1 mV. Mean ± SD, n = 3.

3.3 Synthesis of HL-HBPs with varied particle diameter.

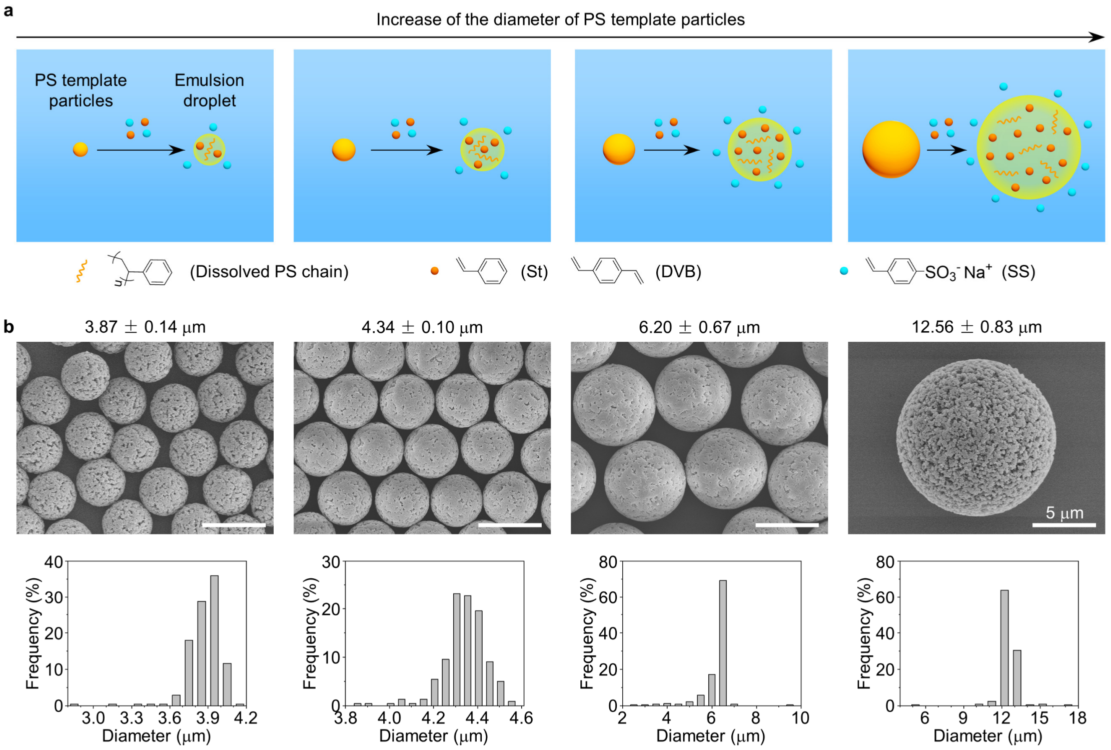

Supplementary Fig. 3. Synthesis and characterizations of HL-HBPs with varied diameter. **a**, HL-HBPs with increased diameter were prepared via emulsion interfacial polymerization by increasing the diameter of polystyrene (PS) template particles. **b**, SEM images and particle diameter distribution histograms of HL-HBPs with diameter of 3.87 ± 0.14 μm, 4.34 ± 0.10 μm, 6.20 ± 0.67 μm, and 12.56 ± 0.83 μm. Mean ± SD, n > 200. For preparation of PSS-PSDVB HL-HBPs with varied diameter, the PS template particles with varied diameter were employed, as well as the feed amount of hydrophobic monomers (St and DVB) was adjusted, with other conditions unchanged as described in the main text. Details are listed as follows. For the synthesis of HL-HBPs with diameter of 3.87 ± 0.14 μm: PS (1.9 μm), St (2 mL), and DVB (1 mL). For the synthesis of HL-HBPs with diameter of 4.34 ± 0.10 μm: PS (2.5 μm), St (0.5 mL), and DVB (1 mL). For the synthesis of HL-HBPs with diameter of 6.20 ± 0.67 μm: PS (2.8 μm), St (0 mL), and DVB (3 mL). For the synthesis of HL-HBPs with diameter of 12.56 ± 0.83 μm: PS (7.2 μm), St (0 mL), and DVB (1 mL).

3.4 Synthesis of HL-HBPs with varied BET surface area.

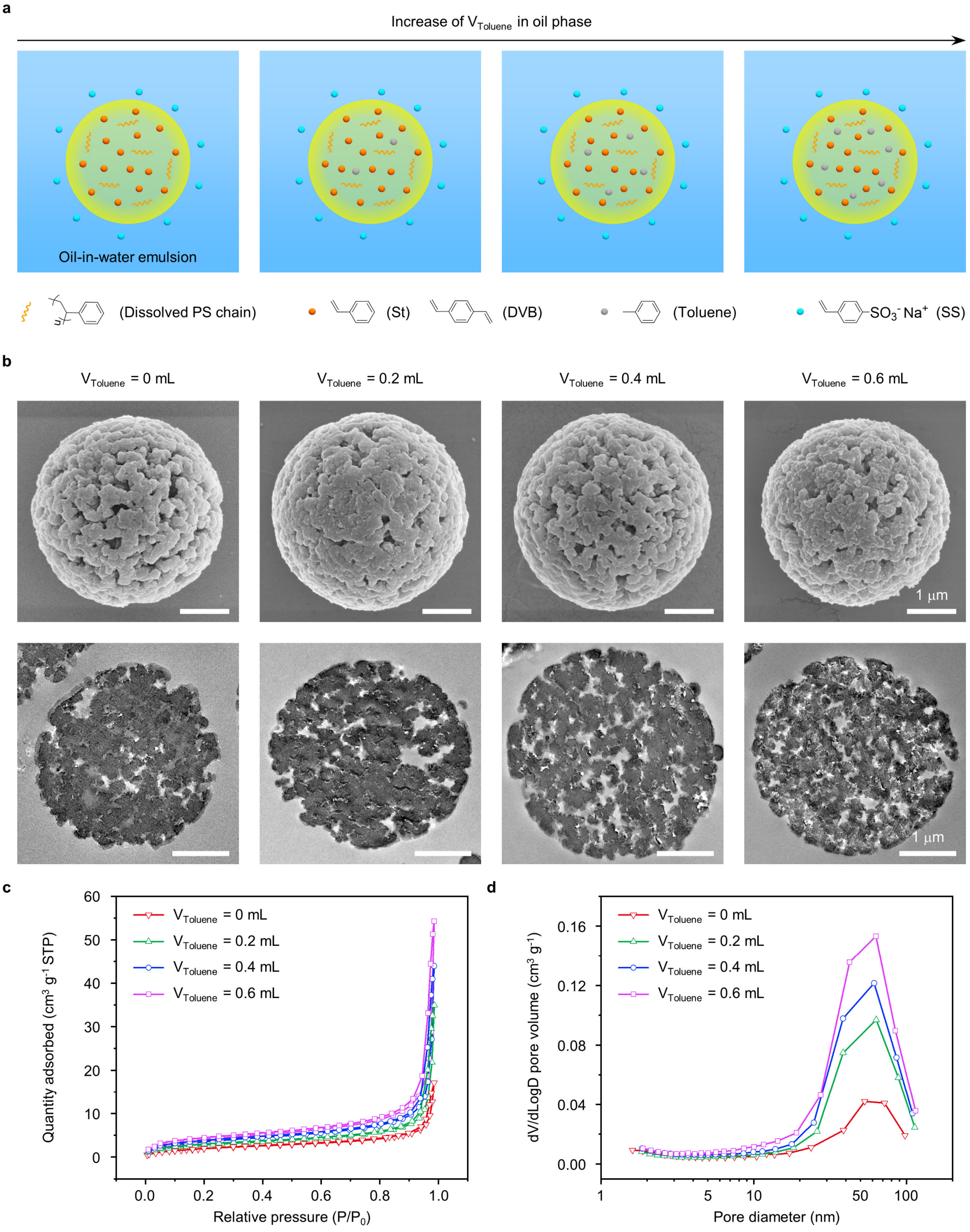

Supplementary Fig. 4. Synthesis and characterizations of HL-HBPs with varied BET surface area. a, HL-HBPs (PSS-PSDVB) with increased BET surface area were prepared via emulsion interfacial polymerization by increasing the addition volume of porogen ($V_{Toluene}$) in oil phase. **b**, SEM images and cross-section TEM images, **c**, Nitrogen adsorption-desorption isotherms, and **d**, Pore size distribution graphs of HL-HBPs with BET surface area of 7.6 ± 0.1 $m^2\ g^{-1}$, 10.2 ± 0.1 $m^2\ g^{-1}$, 13.0 ± 0.1 $m^2\ g^{-1}$, and 14.8 ± 0.1 $m^2\ g^{-1}$ (Mean ± SD, n = 3). The adsorption-desorption isotherms

indicate a type IV adsorption model. Pore size distribution graphs show the HL-HBPs exhibit micropores, mesopores, and macropores. Most of the pores exhibit pore diameter from 10 nanometers to 100 nanometers. $V_{Toluene}$ = 0 mL, 0.2 mL, 0.4 mL, and 0.6 mL, respectively. Other conditions are unchanged as described in the main text. The BET surface area values of the HL-HBPs were obtained via nitrogen adsorption-desorption tests using a physisorption apparatus (Micromeritics, ASAP 2460), before which the particles were degassed at 80 ºC for 8 h.

4. Dispersion performance investigation.

4.1 Characterizations of HLPs and HBPs.

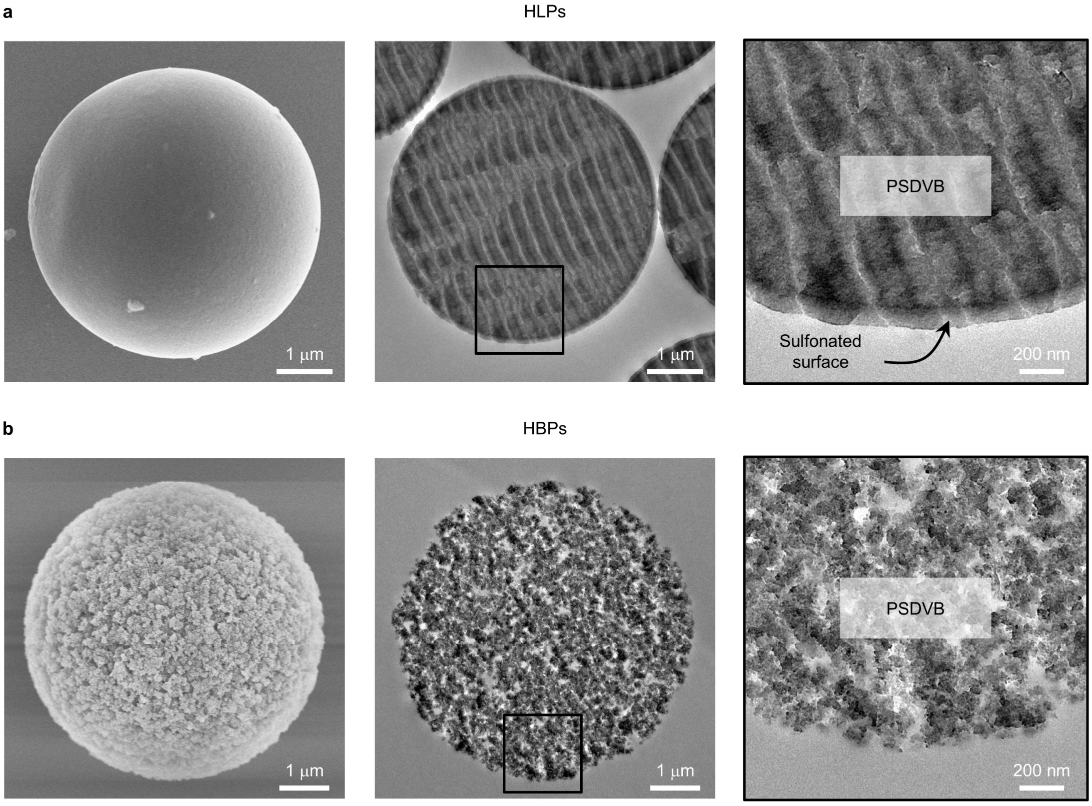

Supplementary Fig. 5. SEM and cross-section TEM images of commercially available HLPs and HBPs. a, HLPs. **b**, HBPs. HLPs are PSDVB particles whose surfaces are modified with hydrophilic sulfonated groups, and HBPs are porous PSDVB particles.

4.2 Hydrophilicity/hydrophobicity nature of particles.

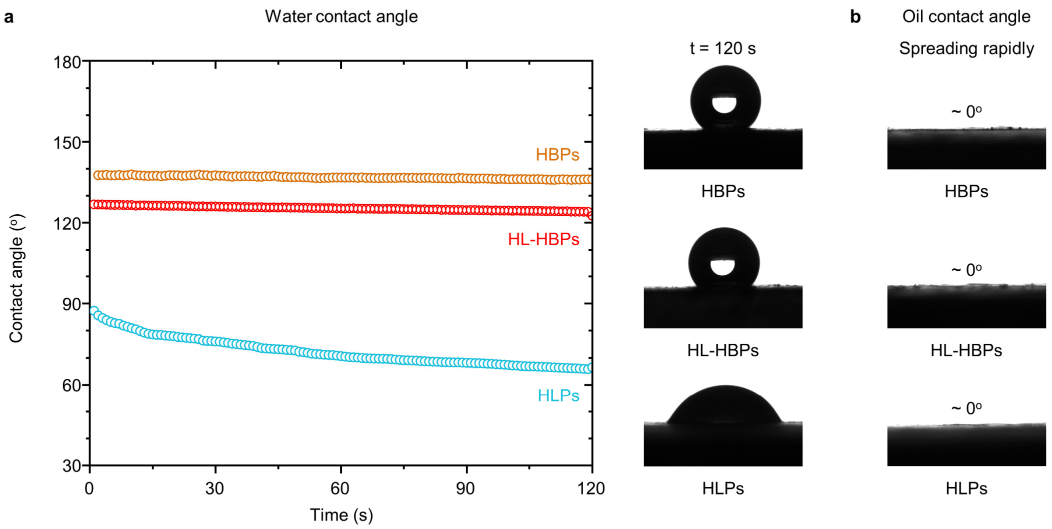

Supplementary Fig. 6. Contact angle measurement of HL-HBPs, HLPs, and HBPs. a, Time-lapse water contact angle measurement in air. The HBPs show a relatively hydrophobic nature, with contact angle of 136.0 ± 2.7° at 120 s. The HLPs show a relatively hydrophilic nature, with contact angle of 66.5 ± 0.4° at 120 s. The HL-HBPs show a relatively hydrophilic nature compared with HBPs, and relatively hydrophobic nature compared with HLPs, with contact angle of 122.6 ± 1.6° at 120 s. These results suggest that the HL-HBPs are more favorable for water wetting compared with HBPs, facilitating their dispersion in water. Contact angle values: Mean ± SD, n = 3. **b**, Oil contact angle measurement in air. The oil droplets spread rapidly for all particles, probably due to the low interfacial tension of the oil (octane). Contact angle measurement process is described as follows. The particle powders were adhered to a tape on a glass slide. Powders that were not tightly adhered were blew away. A drop (2 μL) of water or oil (octane) was dropped on the particles. A surface analyzer (LSA 100, LAUDA Scientific, GmbH) was used to determine the contact angles. For water contact angle in air, the data were automatically calculated and recorded by the software. For oil contact angle in air, the oil droplets can spread rapidly with a contact angle of ~0°. We would like to clarify that the contact angle for HL-HBPs adhered tape (122.6 ± 1.6°) does not certainly imply the absence of electrostatic interaction on HL-HBPs. The hydrophilicity-hydrophobicity of the particles was evaluated by a commonly-used approach. Typically, the particles were adhered to a glass microscope slide by double-sided adhesive tape, and three-phase contact angle was measured by the sessile drop method[1]. The contact angle can be affected by the coverage of the particles on the tape, the interfacial tension of the

particles, and the surface structure[2, 3]. The HL-HBPs adhered tape shows a contact angle of 122.6 ± 1.6^o, which is much reduced compared with HBPs adhered tape (136.0 ± 2.7^o), suggesting the presence of negatively charged hydrophilic domains (PSS).

5. Theoretical calculations for dispersion mechanism explanation.

5.1 Parameters for calculation.

Supplementary Table 2. Parameters of employed materials for calculation.

Material	Dielectric constant (ε_1)	Refractive index (n_1)	Hamaker constant (A, 10^{-20} J)
PSDVB	2.4[4]	1.6[5]	9.601
PSS	2.56[6, 7]	1.5335[a]	7.906
Water	80.2[5]	1.333[5]	3.733
Octane	1.948[5]	1.398[4]	4.759
Air	1.000585	1.0003	3.380×10^{-6}

[a] The value was obtained in Supplementary Fig. 8.

5.2 Demonstration of oil permeation into the HL-HBPs.

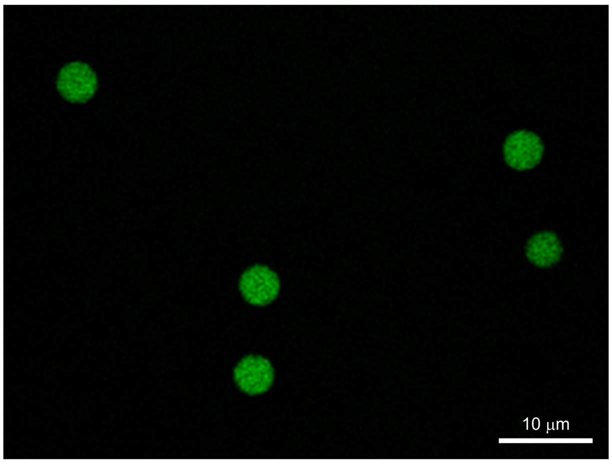

Supplementary Fig. 7. LSCM image of HL-HBPs dispersed in C6 labeled octane solution. The image implies that octane can permeate into the interior of HL-HBPs. The LSCM image was obtained on LSCM instruments affiliated to an optical microscope (Nikon, Eclipse Ti).

5.3 Refractive index prediction of PSS.

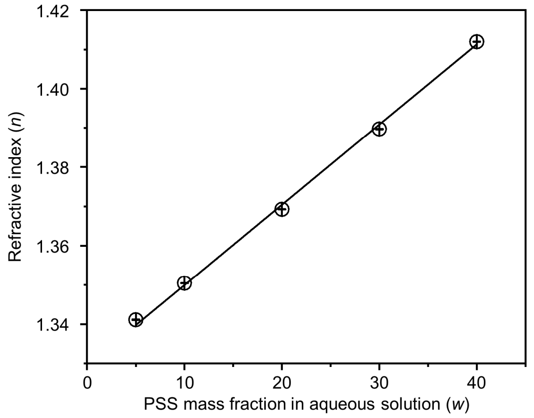

Supplementary Fig. 8. Relationship between refractive index (*n*) and PSS mass fraction (*w*) in aqueous solution. The refractive index (*n*) of pure PSS was predicted by measuring the refractive indices of PSS aqueous solutions with different PSS mass fraction (*w*). A series of PSS (average Mw = 70000, purchased from Sigma Aldrich) aqueous solutions were prepared with PSS mass fraction of 5%, 10%, 20%, 30%, and 40%, respectively. Subsequently, their refractive indices were measured with an Abbe refractometer at 20 °C (*n*20/D) (Mean ± SD, n = 3). The relationship between *n* and *w* can be linearly fitted[8] with an equation of $n = 0.00204 \times w + 1.3295$ ($R^2 = 0.99881$). When pure PSS is applied (w = 100%), n = 1.5335, which is in accordance with previous study[9].

6. Calculation of the interaction sites.

Supplementary Table 3. Calculation details of n_{Sum} of the organic dyes. We make a rough classification of the interaction site according to the group property. (1) Ionic groups, including $-N^+$, $-NH^+$, $-NH_2^+$, and $-SO_3^-$, are regarded as electrostatic interaction site. (2) Polar groups containing hydrogen atoms, including -COOH, -NH-, $-NH_2$, -OH, and $-SO_3H$, are regarded as hydrogen bonding interaction site. (3) Groups containing π-conjugated structures, including benzene ring and naphthalene, are regarded as hydrophobic/π-π bonding interaction site. To simplify the calculation, the protonation/deprotonation of polar groups (such as -COOH, -NH-, $-NH_2$, -OH, and $-SO_3H$) is not taken into consideration. It should be noted that the interactions between materials and complex dyes are difficult to understand comprehensively. In this part, we attempt to use adsorption index as a rough parameter to understand the difference of adsorption amounts for various organic dyes.

Dye	Electrostatic interaction site	Hydrogen bonding interaction site	Hydrophobic/π-π bonding interaction site	n_{Sum}
MG	1 ($-N^+$)	0	3 (Benzene ring)	4
MV	1 ($-NH^+$)	0	3 (Benzene ring)	4
RB	1 ($-N^+$)	1 (-COOH)	3 (Benzene ring)	5
CV	1 ($-N^+$)	0	3 (Benzene ring)	4
MLB	1 ($-N^+$)	0	2 (Benzene ring)	3
MB	2 ($-SO_3^-$)	3 (1 ($-SO_3H$), 2 (-NH-))	6 (Benzene ring)	11
AF	2 ($-SO_3^-$)	2 ($-NH_2$)	3 (Benzene ring)	7
CR	2 ($-SO_3^-$)	2 ($-NH_2$)	4 (2 (Benzene ring), 2 (Naphthalene))	8
EB	4 ($-SO_3^-$)	2 (2 adjacent $-NH_2$/-OH on naphthalene)	4 (2 (Benzene ring), 2 (Naphthalene))	10
MO	1 ($-SO_3^-$)	0	2 (Benzene ring)	3

7. Dye adsorption and desorption performance of HL-HBPs.

7.1 Comparison of the dye adsorption performance.

Supplementary Table 4. Comparison of the pore size, BET surface area, dye adsorption capacity, dye adsorption equilibrium time/kinetics, initial adsorbent dosage, and initial dye dosage of HL-HBPs with materials reported in literatures.

Materials	Pore size	BET surface area	Adsorption capacity	Adsorption equilibrium time/kinetics	Initial adsorbent dosage	Initial dye dosage	Ref.
MOFs ($[In_3O(COO)_6]^+$-based)	0.28 nm~1.37 nm	1078 m^2 g^{-1}	20:1 in molar ratio (MOF:dye)	16 h~64 h	~2.2 mg mL^{-1}	~35 ppm (~7.9×10^{-5} M)	10
MOFs (NH_2-UiO-66)	~1 nm	1035 m^2 g^{-1}	Up to 697.7 mg g^{-1}	Langmuir K_L: 0.006 g mg^{-1} min^{-1}	0.5 mg mL^{-1}	100 ppm	11
Activated carbons (from woods)	/	/	~10 mg g^{-1}	45 min	3.2 mg mL^{-1}	50 ppm	12
Activated carbons (Calgon, USA)	1.078 nm~1.088 nm	972 m^2 g^{-1}~1015 m^2 g^{-1}	Up to 1.4 mmol g^{-1}	> 700 h	0.025 mg mL^{-1}	1.2×10^{-5} M~4.6×10^{-5} M	13
Ferromagnetic hierarchical porous carbon	Wide range	260 m^2 g^{-1}	0.16 mg m^{-2}	~2 h	1 mg mL^{-1}	1.5×10^{-4} M	14
Carbon particles	1~10 nm	5.2 m^2 g^{-1}	Up to 79.5 mg g^{-1}	10 s	0.25 mg mL^{-1}	10, 30 ppm	15
Covalent organic polymers	0.51 nm, 0.76 nm, 1.36 nm	479 m^2 g^{-1}	/	30 min	1 mg mL^{-1}	5×10^{-5} M	16
LDHs (MgAl-LDH)	/	/	Up to 186 mg g^{-1}	5 min	/	100 ppm	17
LDHs (NiAl-LDH)	3.4 nm	97 m^2 g^{-1}	150 mg g^{-1}	6 min	1 mg mL^{-1}	150 ppm	18

Carbon-doped boron nitride	/	18.7 m^2 g^{-1}	Up to 747.10 mg g^{-1}	2 h~3 h	0.2 mg mL^{-1}	150 ppm	19
MgO	/	154.85 m^2 g^{-1}	Up to 549.45 mg g^{-1}	> 20 min	/	100 ppm	20
Chitosan hydrogel	4.34 nm~7.10 nm	2.15 m^2 g^{-1}~42.67 m^2 g^{-1}	Up to 1836 mg g^{-1}	> 300 min	/	450 ppm	21
Amino grafted MCM-41	~3.4 nm	/	Up to 300 mg g^{-1}	> 5 min	0.2~6 mg mL^{-1}	10, 50 ppm	22
HL-HBPs	1 nm~100 nm	7.6 m^2 g^{-1}~14.8 m^2 g^{-1}	Up to 13.62 mg g^{-1}	5 s	0.67~6.67 mg mL^{-1} (total)	10 ppm (2.1×10^{-5} M, RB) (total)	This work

7.2 Desorption of dyes from HL-HBPs characterized by LSCM.

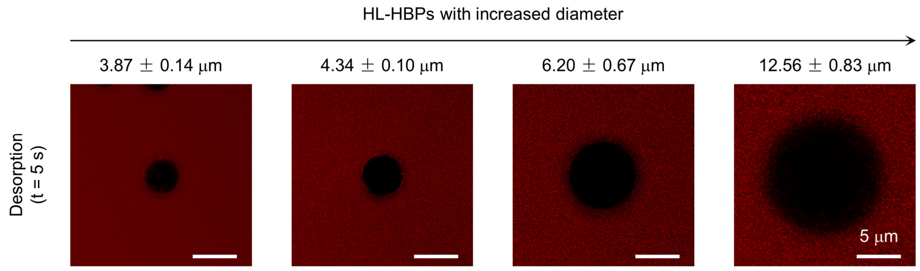

Supplementary Fig. 9. Desorption of RB from PSS-PSDVB HL-HBPs with varied diameter. LSCM images show that RB can be rapidly desorbed (desorption time: 5 s) from all HL-HBPs after adding the organic solvent. Dye desorption experiment was performed as follows. Equal volume of particle suspension (PSS-PSDVB, 1.33 mg mL^{-1}) and RB aqueous solution (20 ppm) were mixed at room temperature, and maintained for 1 h. Subsequently, the aqueous solution was separated by a filter paper (pore size, 2 μm). Then the obtained PSS-PSDVB HL-HBPs adsorbed with RB were dispersed in a mixture of ethanol and n-hexane (1:1 v/v) to achieve dye desorption, and maintained for 5 s. Then 10 μL of the mixed organic solution was encapsulated between two cover glass slides for LSCM visualization. Diameter: Mean ± SD, n > 200.

7.3 Dye adsorption performance of HL-HBPs at different pH.

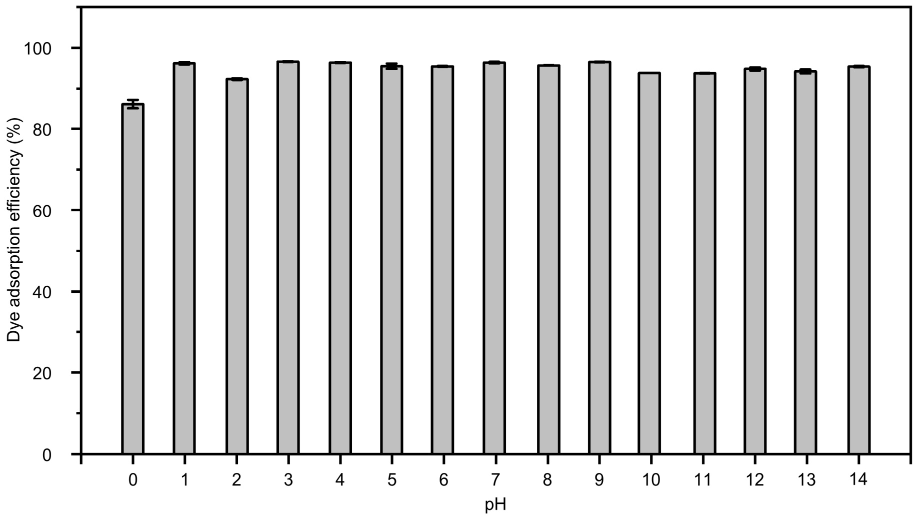

Supplementary Fig. 10. Adsorption of RB with HL-HBPs at pH range of 0-14. The dye adsorption efficiency maintains >92% at pH range of 1-14 without significant decrement. When pH = 0, the dye adsorption efficiency slightly decreases to 86.15 ± 1.09% (Mean ± SD, n = 3). These results indicate the dye adsorption can be achieved in a wide range of pH value.

7.4 Comparison of dye adsorption kinetics.

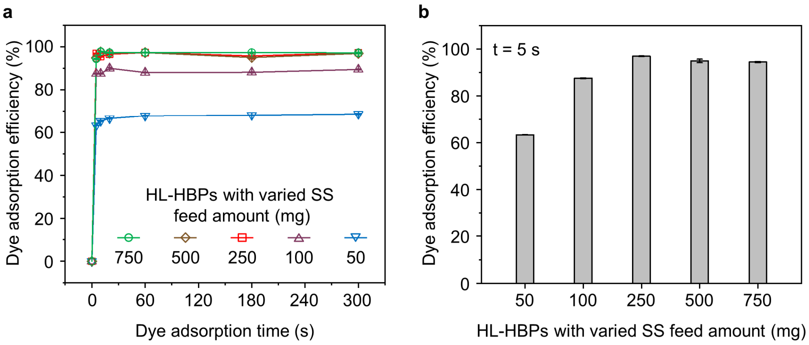

Supplementary Fig. 11. Comparison of dye adsorption kinetics for HL-HBPs prepared with different feed amount of negatively charged hydrophilic monomer. **a**, Dye adsorption efficiency of HL-HBPs with varied SS feed amount at different time. Mean ± SD, n = 3. **b**, Dye adsorption efficiency of HL-HBPs with varied SS feed amount at adsorption time of 5 s. Mean ± SD, n = 3. The feed amount of SS (50, 100, 250, 500, and 750 mg) was varied in the emulsion interfacial polymerization process for the synthesis of HL-HBPs. Resultant HL-HBPs were compared for dye adsorption efficiency at different adsorption time. For all HL-HBPs, the dye adsorption efficiency was almost achieved in 5 s. As the SS feed amount increases from 50 mg to 250 mg, dye adsorption efficiency of corresponding HL-HBPs increases at adsorption time of 5 s. Further increasing the feed amount of negatively charged hydrophilic monomer (SS) to 500 mg and 750 mg has little influence on the dye adsorption efficiency. These results indicate that the dye adsorption is highly dependent on the negatively charged hydrophilic domain (PSS) content. These hydrophilic domains could provide intense electrostatic field for strong electrostatic interaction, facilitating rapid dye adsorption.

7.5 Property changes of HL-HBPs after adsorption-desorption.

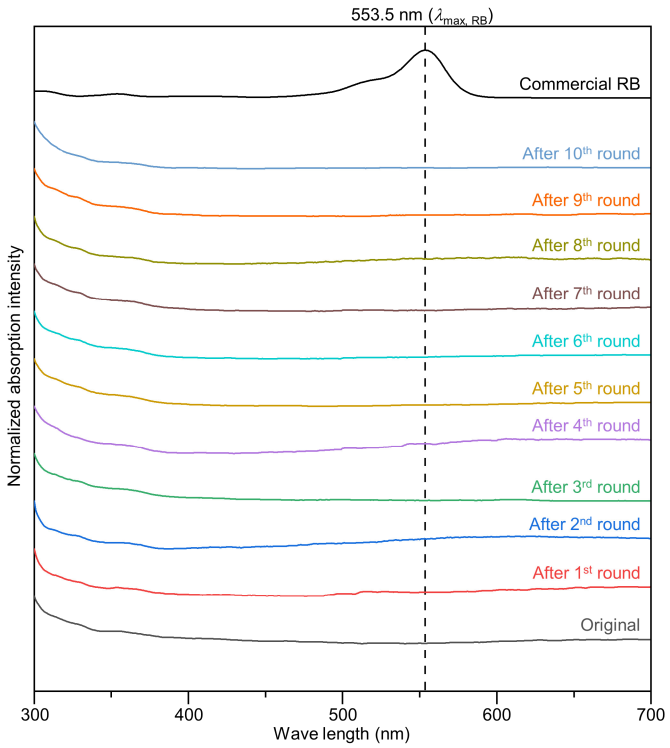

Supplementary Fig. 12. UV-vis absorption spectra of HL-HBPs before and after adsorption-desorption process. The absorption spectra have rarely changed after 10 rounds of adsorption-desorption process, including the characteristic absorption peak of the dyes ($\lambda_{max,RB}$ = 553.5 nm), indicating that dyes are rarely retained on the HL-HBPs. The UV-vis absorption spectra measurements were performed on a microplate reader (Agilent, EPOCH2, USA). HL-HBPs, either original or those obtained after 1st-10th adsorption-desorption process, were dispersed in water for UV-vis absorption spectra measurements from 300 nm to 700 nm.

7.6 Property changes of dyes after adsorption-desorption.

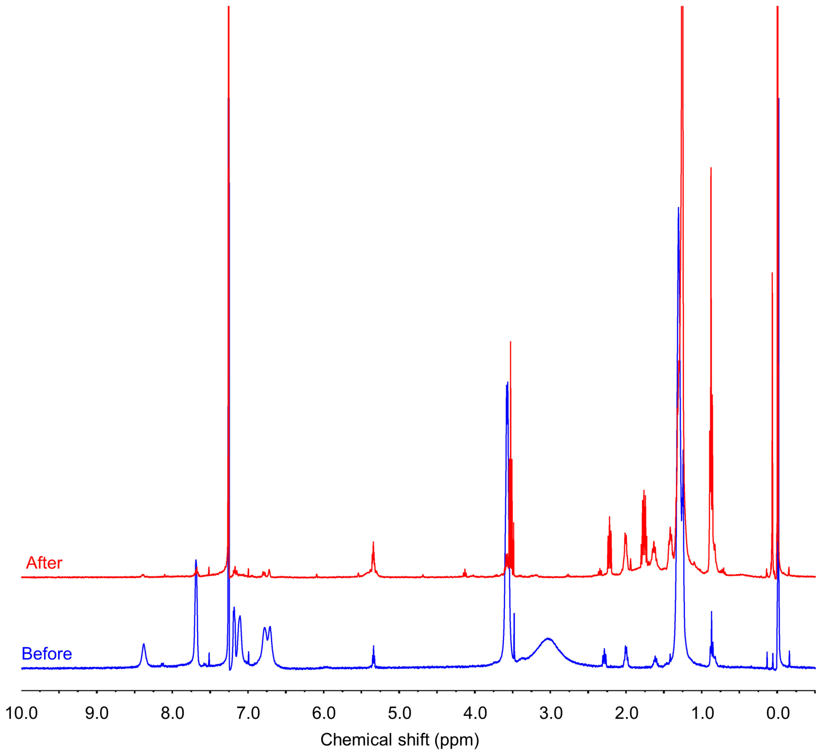

Supplementary Fig. 13. ^{1}H NMR spectra of dyes (RB) before and after adsorption-desorption process. The chemical shift positions have rarely changed after the adsorption-desorption process, indicating that there are rare impurities in the recycled dyes. The NMR measurements were performed on a Ascend^{TM} 400 spectrometer (400 MHz, Bruker, Switzerland). Dyes, either before or after adsorption-desorption process, were dissolved in $CDCl_3$ for ^{1}H NMR measurement.

7.7 Property changes of membranes after filtration.

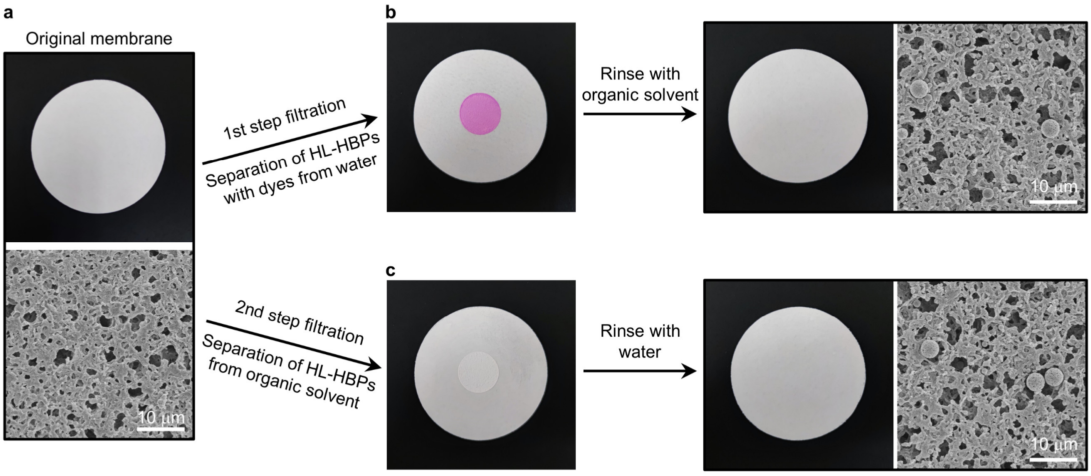

Supplementary Fig. 14. Photos and SEM images of original membranes and membranes after 1st step and 2nd step filtration. a, The original membranes are composed of nylon (polyamide), with average pore diameter of 2 µm. Such pore size allows the fast flow of water and retention of particles. **b**, The HL-HBPs adsorbed with dyes are retained on the membrane after 1st step filtration. Most HL-HBPs can be rinsed out using organic solvent, leaving minority of them retained on the membranes. **c**, The HL-HBPs are retained on the membrane after 2nd step filtration. Most HL-HBPs can be rinsed out using water, leaving minority of them retained on the membranes. The total recovery ratio of HL-HBPs is about 95.5%.

8. Large scale preparation of HL-HBPs.

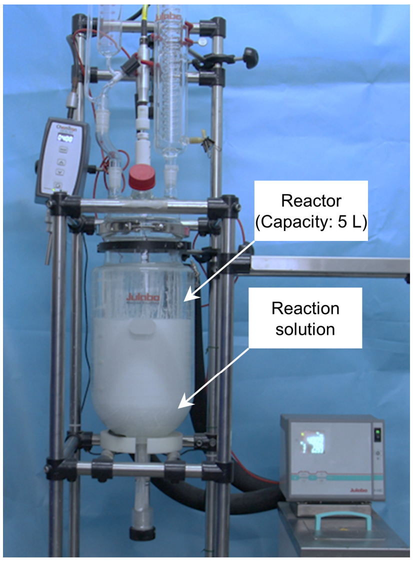

Supplementary Fig. 15. Photograph showing a 5 L reactor for large scale synthesis of HL-HBPs.

9. Large scale dye recycle and wastewater treatment using HL-HBPs.

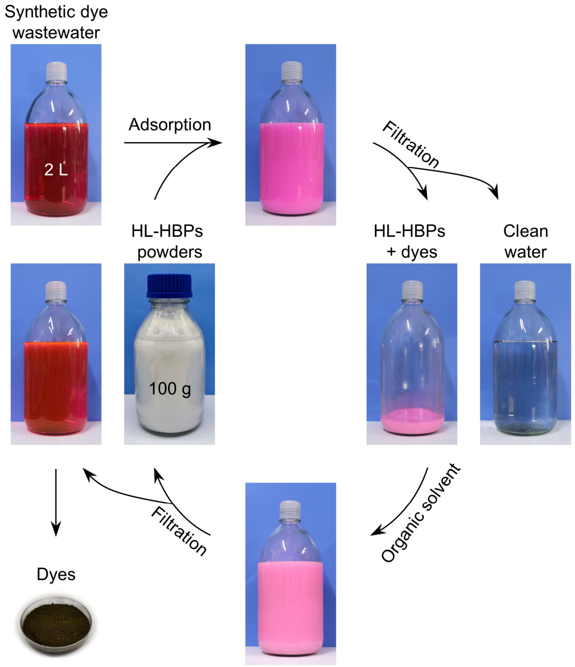

Supplementary Fig. 16. Photographs showing the adsorption and recycle of organic dyes (RB) from 2 L of dye wastewater using HL-HBPs (PSS-PSDVB).

10. Summary of solvent property.

Supplementary Table 5. Summary of the boiling point and polarity of various organic solvents that are potential for the desorption of organic dyes.

Solvent	Boiling point (ºC)	Polarity
ACN	82	6.2
Ethanol	78	4.3
EA	77	4.3
TCM	77	1.6
n-hexane	69	0.06
THF	66	4.2
Methanol	65	6.6
Acetone	57	5.4
DCM	40	3.4
n-pentane	36	0
EE	35	2.9

11. Comparison of dye recycle performance.

Supplementary Table 6. Comparison of the dye recycle performance for HL-HBPs and materials in literatures. To desorb organic dyes from materials reported in literatures, eluents containing inorganic acid, alkaline, or salt, are often added into the aqueous solution to weaken the interactions between materials and dyes. Such eluents make the dye recycle more complicated. In comparison, organic solvent is used for dye desorption, and dyes can be recycled under a simple distillation process.

Materials	Recyclability of materials	Recyclability of dyes	Eluents	Ref.
MOFs	Yes	Not available	Salt ($NaNO_3$)	10, 11
LDHs	Yes	Not available	Salt containing Cl^-, NO^{3-}, or CO_3^{2-}	17, 18
Carbon-doped boron nitride	Yes	Not available	Acid (HCl)	19
MgO	Yes	Yes	Acid (HCl)	20
Chitosan hydrogel	Yes	Not available	Alkali (NaOH)	21
Amino grafted MCM-41	Yes	Not available	Alkali (NaOH)	22
HL-HBPs	Yes	Yes	Organic solvent	This work

References

1. Nowak, E., Combes, G., Stitt, E.H. & Pacek, A.W. A comparison of contact angle measurement techniques applied to highly porous catalyst supports. *Powder Technol.* **233**, 52-64 (2013).
2. Gao, N. & Yan, Y. Characterisation of surface wettability based on nanoparticles. *Nanoscale* **4**, 2202-2218 (2012).
3. Zhou, X., Guo, X., Ding, W. & Chen, Y. Superhydrophobic or superhydrophilic surfaces regulated by micro-nano structured ZnO powders. *Appl. Surf. Sci.* **255**, 3371-3374 (2008).
4. Brandrup, J. Polymer handbook (4th edition). (John Wiley and Sons, 1999).
5. Speight, J.G. Lange's handbook of chemistry. (The McGraw-Hill Companies, Inc., 2004).
6. Zong, Y., Xu, F., Su, X. & Knoll, W. Quartz crystal microbalance with integrated surface plasmon grating coupler. *Anal. Chem.* **80**, 5246-5250 (2008).
7. Sheng Hsiung, C., Chien-Hung, C., Feng-Sheng, K., Chuen-Lin, T. & Chun-Guey, W. Unraveling the enhanced electrical conductivity of PEDOT:PSS thin films for ITO-free organic photovoltaics. *IEEE Photonics J.* **6**, 1-7 (2014).
8. Roots, J. & Nyström, B. Concentration and temperature dependence of the refractive index increment of a polystyrene sample in trans-decalin. *J. Polym. Sci. Polym. Phys. Ed.* **16**, 695-701 (1978).
9. Ono, Y., Nakase, I., Matsumoto, A. & Kojima, C. Rapid optical tissue clearing using poly(acrylamide-co-styrenesulfonate) hydrogels for three-dimensional imaging. *J. Biomed. Mater. Res. B Appl. Biomater.* **107**, 2297-2304 (2019).
10. Zhao, X. et al. Selective anion exchange with nanogated isoreticular positive metal-organic frameworks. *Nat. Commun.* **4**, 2344 (2013).
11. Wang, H. et al. Membrane adsorbers with ultrahigh metal-organic framework loading for high flux separations. *Nat. Commun.* **10**, 4204 (2019).
12. Heibati, B. et al. Kinetics and thermodynamics of enhanced adsorption of the dye AR 18 using activated carbons prepared from walnut and poplar woods. *J. Mol. Liq.* **208**, 99-105 (2015).
13. Wang, S. & Zhu, Z. Effects of acidic treatment of activated carbons on dye adsorption. *Dyes Pigments* **75**, 306-314 (2007).
14. Wang, D.-W., Li, F., Lu, G.Q. & Cheng, H.-M. Synthesis and dye separation

performance of ferromagnetic hierarchical porous carbon. *Carbon* **46**, 1593-1599 (2008).

15. Seifikar, F., Azizian, S. & Sillanpaa, M. Microwave-assisted synthesis of carbon powder for rapid dye removal. *Mater. Chem. Phys.* **250**, 123057 (2020).
16. Byun, J., Patel, H.A., Thirion, D. & Yavuz, C.T. Charge-specific size-dependent separation of water-soluble organic molecules by fluorinated nanoporous networks. *Nat. Commun.* **7**, 13377 (2016).
17. Sansuk, S., Srijaranai, S. & Srijaranai, S. A new approach for removing anionic organic dyes from wastewater based on electrostatically driven assembly. *Environ. Sci. Technol.* **50**, 6477-6484 (2016).
18. Pahalagedara, M.N. et al. Removal of azo dyes: Intercalation into sonochemically synthesized NiAl layered double hydroxide. *J. Phys. Chem. C* **118**, 17801-17809 (2014).
19. Wang, P., Wang, P., Guo, Y., Rao, L. & Yan, C. Selective recovery of protonated dyes from dye wastewater by pH-responsive BCN material. *Chem. Eng. J.* **412**, 128532 (2021).
20. Cao, N. et al. Superior selective adsorption of MgO with abundant oxygen vacancies to removal and recycle reactive dyes. *Sep. Purif. Technol.* **275**, 119236 (2021).
21. Liu, Y. et al. Efficient removal and recycle of acid blue 93 dye from aqueous solution by acrolein crosslinked chitosan hydrogel. *Colloids Surf. Physicochem. Eng. Aspects* **632**, 127825 (2022).
22. Rizzi, V. et al. Amino grafted MCM-41 as highly efficient and reversible ecofriendly adsorbent material for the Direct Blue removal from wastewater. *J. Mol. Liq.* **273**, 435-446 (2019).
